# *Cis*-regulatory decoy disrupts autorepression: a potential escape-resistant anti-viral therapy

**DOI:** 10.1038/s41392-022-01187-5

**Published:** 2022-09-27

**Authors:** Jia Hu, Yuanqin Min, Xiulian Sun

**Affiliations:** Wuhan Institute of Virology, Chinese Academy of Sciences, Center for Biosafety Mega-Science, Wuhan, 430071 China

**Keywords:** Molecular biology, Pathogenesis, Microbiology

**Fig. 1:**
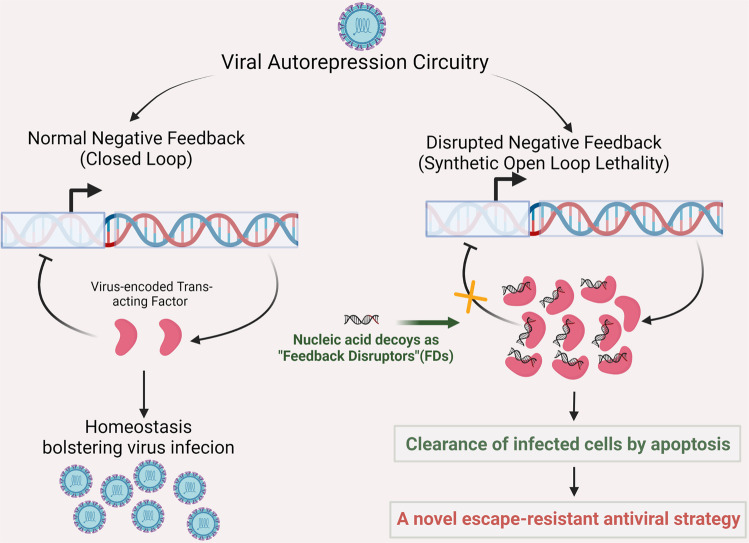
Synthetic “open-loop lethality” makes autorepression circuitry the viral Achilles’ heel. Virus-encoded specific protein can serve as *trans*-acting factor that binds to the *cis-*repression sequence on viral genome and auto-represses viral transcription to avoid aberrant viral protein expression, thus maintaining homeostasis beneficial for virus infection (left). Artificially synthesized nucleic acid decoys (molecular mimics of the *cis*-regulatory binding sites) can disrupt the normal negative feedback, raising viral protein expression to cytotoxic levels and leading to clearance of infected cells by apoptosis (right). This synthetic “open-loop lethality” turns the viral autorepression circuitry into a fatal weakness of virus life cycle, representing a novel and escape-resistant antiviral strategy

In a recent paper, Chaturvedi et al. published a study in *Cell* reporting that the artificially synthesized nucleic acid decoys would disrupt the autorepression, which can generate antiviral agents with a high barrier to the evolution of resistance^1^ (Fig. 1).

Double-stranded transcription factor decoy oligodeoxynucleotides are short double-stranded DNA molecules^2^ that represent a novel class of therapeutic drug candidates.^3^ Transcription factor decoy oligodeoxynucleotides mimic the specific binding site of a target transcription factor in the *cis*-regulatory promoter region. The team focused on a negative-feedback circuitry that regulates viral replication in the herpesvirus immediate early locus. Theoretically, the established models implied that TFD may represent feedback-disruptor (FD) molecules and specifically interrupt immediate early feedback. Previous computational models of ganciclovir resistance evaluated the possibility that viruses with n mutations would develop in vivo in order to estimate the likelihood of resistance to FD compounds. It was shown that the chance of viruses emerging with the required number of mutations to re-specify a DNA-protein interaction scaled with ~µ^−n^.

An in vitro experiment to evaluate the effectiveness of several linear DNA duplexes was designed to help find DNA duplexes that might potentially serve as FDs by binding to IE86. The crs-encoding DNA duplexes of various lengths were tested after the C-terminal portion of IE86 was purified. The results showed that the 28-bp DNA duplex provided maximal binding. To test whether the duplexes disrupted feedback in cells, the IE86 promoter-enhancer was used to create feedback reporter cells that produced both IE86 and GFP. Flow cytometry demonstrated that duplex hindered autorepression and raised IE86 levels. The duplexes were stabilized to enhance feedback disruption by inserting internal phosphorothioate bonds and labeled FD^86^. Then, the viral feedback loop was then demonstrated to be disrupted by the particular DNA duplex to produce synthetic lethality. However, FD^86^ duplexes neither stimulate the DNA sensor nor activate the innate immune or inflammatory pathway.

Subsequently, flow cytometry and microscopy revealed that FD^86^ generated a greater than 10-fold increase in IE86 expression in infected cells. The FD^86^ IC_50_ was 30-fold lower than that of fomivirsen. Furthermore, a viral strain carrying a *crs* point mutation was utilized to demonstrate that FD-induced cell death was caused by the breakdown of IE86 feedback. FD^86^ did not decrease virus replication or increase IE86 expression in the mutant strain, which suggested that FD-mediated viral inhibition was due to the specific disruption of IE86 autorepression. Moreover, it was shown that FD^86^ abundance correlated with the multiplicity of infection, as increased virus would produce more cytotoxic IE86 proteins. The results also revealed that the antiviral effects were not restricted to a specific cell line or strain type. Next, apoptosis markers and a cell death rescue assay were employed to ascertain if viral inhibition was caused by IE86 apoptosis-mediated “open-loop lethality”. The results indicated that FD^86^ produces cell death via apoptosis to reduce viral titers. Similar results suggested that the other herpesvirus circuits may be susceptible to the FD-mediated antiviral approach. A continuous-culture system was employed to examine whether a genetic barrier to the emergence of resistance is presented by open-loop lethality. The team also compared the FD antiviral effects against fomivirsen.^4^ In contrast to fomivirsen, FD^86^ did not develop resistance and the virus was undetectable in the subcultures.

In order to verify that viral titers were decreased as a result of FDs’ long-lasting antiviral action, the culture supernatants were analyzed after the removal of FDs. After the removal of FDs, virus titers recovered by 1.5–2 logs, which demonstrated that the lack of resistance was not due to the early emergence of a reduced-fitness variant that subsequently disappeared. A genotypic resistance assay further confirmed that no resistant variants had emerged. Similar results were detected in other herpesviruses. An established dose-escalation approach was applied to choose the potential FD-resistant mutants. However, no evidence of resistance to FDs was found.

In addition, the team established a bystander co-culture experiment to rule out the possibility of bystander killing and demonstrated that FD-mediated apoptosis was not initiated by bystander cells. Next, they determined that FD duplexes had the potential to be used both individually and in multiplex treatments, both of which would increase the antiviral impact of traditional antivirals.

Moreover, the HSV-1 corneal infection in a well-established mouse model and the murine CMV systemic infection model were employed to test whether open-loop lethality inhibits viral replication in vivo. The results showed that FD treatment significantly reduced the HSV-1 titers in mouse corneas and that the breakdown of feedback, which results in open-loop mortality can provide a systemic antiviral impact.

Next, the study focused on the beta coronavirus SARS-CoV-2 to examine whether the feedback disruption could be generally applied to suppress other viruses, e.g., RNA sarbecoviruses. FD-induced apoptosis was associated with a more than 10-fold decrease in SARS-CoV-2 numbers. According to these findings, breaking up the circuit produces open-loop lethality, which causes an RNA virus to become antiviral.

In summary, the study indicated that transcriptional negative-feedback loops can be disrupted by a cis-regulatory decoy. In addition, feedback disruption increases expression levels, resulting in open-loop lethality, overcoming a major therapy challenge: the decline in antiviral effectiveness that comes with large viral loads for traditional antiviral medications. Finally, open-loop lethality inhibits viral replication in DNA and RNA viruses and feedback disruption targets are widespread.
